# Reliability, validity, and measurement invariance of a Chinese handwriting legibility scale among primary students in central China

**DOI:** 10.3389/fpsyg.2023.1050894

**Published:** 2023-07-28

**Authors:** Hong Lu, Xin Chen, Frederick K. S. Leung, Haode Zuo

**Affiliations:** ^1^Faculty of Education, The University of Hong Kong, Pokfulam, Hong Kong SAR, China; ^2^College of Mathematical Science, Yangzhou University, Yangzhou, Jiangsu, China

**Keywords:** Chinese handwriting, legibility, spatial cognition, development and validation, primary school students

## Abstract

**Background:**

Chinese handwriting has a close relationship with spatial cognition, and the legibility dimension is prominent with its spatial-oriented characteristics. However, handwriting evaluation focusing on the detailed spatial aspects of the legibility dimension in the Chinese context is rare.

**Aims and methods:**

We aimed to develop a Chinese Handwriting Legibility Scale (CHLS) and examine its reliability, validity, and measurement invariance among Chinese primary students of different grades. A total of 684 students aged 8–12 years were recruited from a mainstream primary school in central China and were asked to copy a Chinese template as legibly as possible within 4 min. The developed CHLS was used to assess these students’ legibility performance.

**Results:**

The seven-criteria CHLS favored content validity. The inter-rater reliability was good; however, the scoring instructions need to be refined. Principal component analysis (PCA) revealed a one-factor solution explaining 62.336% of the variance of the seven-criteria CHLS, and confirmatory factor analysis (CFA) confirmed its appropriateness. There was a high internal consistency (*α* = 0.902). In terms of measurement invariance, the factor structures and loadings of the CHLS were consistent across students of different grades; however, significant intercept variations were detected between students of Grades 2 and 4.

**Conclusion:**

CHLS may be effective for evaluating Chinese handwriting legibility performance in the Chinese primary school context in the central region. Students’ Chinese handwriting legibility performance may have developmental specificity in different grades.

## 1. Introduction

As children progress through school, they are expected to write legibly at a reasonable speed. Traditionally, handwriting has been closely associated with keeping up with class work and examinations (Barnett et al., 2018) and has been described as a ‘low-level’ perceptual motor skill in the broader writing process (Berninger et al., 2002; Barnett et al., 2018). However, handwriting activities, which dynamically integrate perceptual motor, cognition, tactile, and kinesthetic sensitivities (Feder and Majnemer, 2007), also involve high-level cognitive processes (Kao, 2000). The fact that the “central” cognitive processes and “peripheral” motor processes continuously interact during written word production (Berninger and Swanson, 1994; Graham and Weintraub, 1996; Purcell et al., 2011; Roux et al., 2013; Kandel et al., 2017; Zhang and Feng, 2017) supports this standpoint. Cognitive processes here generally refer to cognitive planning, working memory processes, and phonological and orthographic coding (e.g., McCutchen, 2000; Volman et al., 2006). Whereas motor processes denote the retrieval/production of written forms, planning and ordering of the sequence of letters/characters and execution of specific motor programs (Ellis, 1982). In the field of education, handwriting maneuvers are reportedly intertwined with domain-specific spatial cognition, such as mental rotation (e.g., Li et al., 1999, 2014; Sakamoto and Spiers, 2014), spatial visualization (e.g., Kao, 2000; Likhanov et al., 2018), visual motor integration (e.g., Maeland, 1992; Tseng and Murray, 1994; Weil and Cunningham Amundson, 1994), and visual–spatial working memory (e.g., Flaherty and Connolly, 1995; Demetriou et al., 2005; Kazi et al., 2012). The underlying rationale is that written scripts are rich in geometric patterns and visual–spatial features, and handwriting production requires the script components to be organized with appropriate proportioning and accurate spatial relationships (Lai, 2008). In this sense, owing to the limited working memory capacity of humans, handwriting automation is vital not only for meeting the needs of examinations and daily learning but also for its potential associations with spatial cognition.

Spatial cognition particularly permeates Chinese handwriting processing (Kao, 2000). Compared with alphabetic letters built on phonemic structures, Chinese characters are more thorough visual characters, relying on various visual configurations, and are typically described as a logographic system (Lai, 2008; Lam et al., 2011). Chinese characters possess a high nonlinear complexity, with all strokes and radicals packed into an imaginary uniformed square (Chow et al., 2003; Tan et al., 2005). Thus, Chinese characters demand a substantially higher visual discrimination of fine changes in the shapes, locations, and spatial arrangement of strokes (Tan et al., 2001; Chow et al., 2003; Lai, 2008). Specifically, to legibly write a Chinese character, the writer needs to pay visual attention to not only the spatial position of each stroke relative to the imaginary square frame but also to the spatial relationship among strokes. After gaining a certain perceptual understanding and memory of the complex spatial relations, the writer then needs “to mentally represent each stroke spatially, to copy each one accurately, and to learn the spatial relations by heart through practice” (Li and Nuttall, 2001, p. 16). According to Li et al. (1999), writing Chinese characters (rather than alphabetic letters, the processing of which is primarily linear and emphasizes smoothness and continuity; Lai, 2008) can provide learners with more opportunities to experience spatial relations in the Euclidean system. Besides, as suggested by the Chinese-character writing’s psycho-geometric theory (Kao, 2000), Chinese handwriting is not only an external projection and execution of the writer’s internal cognitive images of Chinese words but also functions to re-train and improve the writer’s visuospatial cognition. Numerous cross-cultural comparative studies uncovered that East Asian (e.g., Chinese, Japanese) students presented advantage in spatial abilities relative to their Western (e.g., North American and European) counterparts (e.g., Flaherty and Connolly, 1995; Li et al., 1999; Demetriou et al., 2005; Kazi et al., 2012; Sakamoto and Spiers, 2014) and claimed that the differences between the Chinese and alphabetic languages, especially *the writing systems and handwriting processes*, play a fundamental role in shaping this cognitive difference, providing empirical support for this theory.

Although not specified, the close association between Chinese handwriting and spatial cognition is mainly sourced from the dimension of handwriting legibility. Handwriting legibility refers to the clarity, quality, precision, and accuracy of a person’s handwriting production (e.g., Maeland, 1992; Gilboa et al., 2010); according to Tomchek and Schneck (2006, chap. 14), it represents “the degree of the handwriting produced in consideration of alignment and size on a line and spacing between letters and words in relation to each other as well as the organization of the whole page.” As Bo et al. (2014) noted, among the different dimensions of handwriting performance, handwriting legibility reflects more about the spatial characteristics of script layout, relative to handwriting speed and pressure which correspond more to the temporal-oriented characteristics of the handwriting process. Common indicators to operationalize handwriting legibility include spacing/spatial relationships (or spatial organization), size, formation, alignment, slant/direction, and baseline orientation (e.g., Volman et al., 2006; Parush et al., 2010; Rosenblum et al., 2010; Klein et al., 2011; Bo et al., 2014; Linda et al., 2014). These indicators imply that spatial thinking underlies the legible Chinese handwriting processing, highlighting the need to evaluate Chinese handwriting legibility based on its spatial-oriented characteristics. However, despite the significance of this handwriting performance and the fact that numerous students struggle with this area (Lam et al., 2011), little research has been done to concretize the scenarios of Chinese handwriting legibility. This is partly attributable to the absence of robust assessment tools. Most handwriting evaluation scales have been developed to assess the handwriting quality or to detect dysgraphia via the teacher’s overall impressions of children’s handwriting production in class (e.g., Tseng, 1993; Chang and Yu, 2005, 2012; Barnett et al., 2007, 2013, 2018; Rosenblum and Livneh-Zirinski, 2008). These scales provide a valuable overview of various aspects of handwriting, including, for example, global legibility, production speed, page layout, effort to read the script, the motivation/attitude to write, fatigue, writing alterations (attempts made to rectify the writing), pencil grip, and gross movement. However, originating from teachers’ overall impression, these evaluations are often highly subjective, scale poorly, and fail to afford an in-depth analysis of specific, especially spatial, aspects of handwriting performance.

Computerized assessments using digitizing tablets (with various supporting programs, e.g., Rosenblum et al., 2006; Rosenblum and Livneh-Zirinski, 2008; Li-Tsang et al., 2011, 2013; Lee et al., 2016; Mekyska et al., 2016; Pagliarini et al., 2017; Asselborn et al., 2018; Gargot et al., 2020) support the precise measurement of some spatial characteristics (size, spacing, etc.) of handwriting production across diverse handwriting tasks varying in cognitive demands and task lengths. Particularly, combining the temporal characteristics collected during handwriting processing, these technologies make the analysis of legibility, dynamics (e.g., velocity, acceleration, etc.), pressure and even pen tilt of handwriting product and process sophisticated and automatic, thus providing more comprehensive and quantitative information concerning students’ handwriting acquisition and performance/disabilities. This information includes but is not limited to the pattern identification of potential future handwriting impairments at a very early age (e.g., Pagliarini et al., 2017), and the classification (or digital diagnosis) of dysgraphic children (e.g., Mekyska et al., 2016; Asselborn et al., 2018) even with the age effect considered (e.g., Gargot et al., 2020). However, despite the increasing availability of digital tablets and their advantages in dynamic evaluation, regarding legibility evaluation, these new technologies usually pay more attention to stroke accuracy but are limited in holistic legibility. In addition, they are not always accessible to students in regular classrooms, and their manipulation is not intuitive to teachers (Barnett et al., 2018). All these limitations determine that their application to legibility evaluation in the classroom context remains to be improved.

Several scales were developed to assess handwriting legibility in great detail using multiple criteria. Examples of such scales include Minnesota Handwriting Assessment (MHA; Reisman, 2004), Scale of Children’s Readiness In PrinTing (SCRIPT; Weil and Cunningham Amundson, 1994), Hebrew Handwriting Evaluation (HHE; Erez et al., 1996), Concise Assessment Scale for Children’s Handwriting (BHK; Hamstra-Bletz et al., 1987), the Persian Handwriting Assessment Tool (PHAT; Havaei et al., 2017), the Evaluation Tool of Children’s Handwriting-Manuscript (ETCH-M; Amundson, 1995), and Tseng Handwriting Problem Checklist (Tseng, 1993). The specific criteria assessed by these scales include but are not limited to spacing/spatial relationships (the relative position of strokes/letters/characters), the size of strokes/letters/characters, stroke/letter/character formation (e.g., closure, superfluous/missing strokes, line quality, and slant/direction), alignment of letters/characters, and baseline orientation (e.g., out of grid/line, overshooting or undershooting the baseline, and inappropriate margins) (e.g., Volman et al., 2006; Parush et al., 2010; Rosenblum et al., 2010; Klein et al., 2011; Cheng-Lai et al., 2013; Bo et al., 2014; Linda et al., 2014). The consistency/uniformity of or variation/error in the writers’ production relative to a standard typically represents the extent of legibility (Graham et al., 2006; Bo et al., 2014). Although such scales can appropriately assess the spatial characteristics of handwriting production, the extensive list of criteria covered by these scales makes their use in a classroom setting difficult and time-consuming (e.g., Asselborn et al., 2018). Besides, most of these criteria are applicable to alphabetic contexts, and their appropriateness in and contribution to Chinese handwriting legibility assessment are yet to be examined (Li-Tsang et al., 2013) (see a summary of existing handwriting evaluation instruments in Supplementary material).

In this context, both teachers and students need a tailored practical tool for evaluating the detailed spatial aspects of Chinese handwriting legibility. This tool may be helpful in describing and quantifying students’ Chinese handwriting legibility performance and identifying Chinese handwriting difficulties that are specifically caused by spatial cognition deficits, which in turn assisting the development of corresponding supporting plans. This will also enable teachers and students to consider the relationships between Chinese characters and Chinese handwriting and spatial thinking, fostering their consciousness and autonomy of understanding Chinese character learning from a higher-level perspective. Accordingly, in this study, we aimed to develop a reliable and valid Chinese Handwriting Legibility Scale (CHLS) focusing on the detailed spatial aspects of the handwriting legibility dimension.

This study included Chinese students (aged 8–12 years) attending a primary school in central China who had undergone several years of Chinese handwriting training and should have developed certain written communication skills. The students were from Grades 2, 4, and 6, which aligns with the main student groups assessed in related research (e.g., Tseng and Chow, 2000; Li-Tsang et al., 2013; Lee et al., 2016). This cross-grade sampling design was adopted not only because it supports the scale development and validation aiming for a broad application in primary school stages but also because it provides opportunities for examining the measurement invariance of the CHLS (of course constrained by central China). To the researchers’ knowledge, measurement invariance of handwriting instruments has rarely been assessed in previous studies; hence, our study may provide novel insights into the generalizability of the CHLS and the possible developmental changes underlying the Chinese handwriting legibility performance among students of different grades in central China.

## 2. Methods

### 2.1. Development of the CHLS criteria and assessment of content validity

Following the guidelines of Li-Tsang et al. (2013), the measurement criteria were developed or selected based on the following four major concerns: (i) the criteria regularly adopted by schoolteachers or occupational therapists to evaluate students’ (Chinese) handwriting, (ii) the recognized characteristics of children with handwriting problems, (iii) the logographic nature and visual–spatial properties of Chinese characters, and (iv) a review of the literature. Besides, the assessment was based on analytic and holistic impressions of handwriting production suggested by Lam et al. (2011) and Li-Tsang et al. (2013). The analytic evaluation approach focuses on judging or grading the quality of stroke-level (i.e., *within* character) handwriting features according to predetermined standards. By contrast, the holistic evaluation approach focuses on assessing the character-level (i.e., *between* characters) features of a written passage as compared with a group of pre-graded writing samples.

Accordingly, 10 legibility criteria were initially established to form the basis of CHLS: h1: spacing/spatial relationships between *strokes/radicals*; h2: spacing/spatial relationships between *characters*; h3: alignment of characters; h4: baseline orientation; H5: uniformity of *stroke/radical* size; h6: uniformity of *character* size; h7: number of strokes (no superfluous/missing strokes); h8: closure of stroke/radical; h9: line formation; and h10: direction. The instructions for scoring h1, h5, h7, h8, h9, and h10 emphasized decision at the analytic level, whereas those for scoring h2, h3, h4, and h6 corresponded to the legibility performance at the holistic level. A Likert scale ranging from 1 (poor) to 5 (good) with scoring instructions and examples was developed to assess each criterion. These were applied to ‘copying’ products gathered as part of the Smart Handwriting Analysis and Recognition Platform (SHARP) handwriting task assessment (Li-Tsang et al., 2022; see Section 2.5.1 below). China does not have a single, prescribed writing style; however, the basic horizontal and vertical stroke requirements tend to be invariant throughout students’ development, as reflected in our study. The CHLS was applied to the writing with at least three lines of handwriting. The total scores of the initial version ranged from 10 to 50, with higher scores indicating better legibility. A scoring sheet was designed, with the summed score representing the global legibility score. The first author and two research assistants independently scored 10 handwriting samples from Grade 4. This process refined the scoring sheet’s wording and layout to enhance the overall ease of use.

Next, four experts from different professions (Chinese teaching, educational psychology, and mathematics education) evaluated the tool independently. Among them, two Chinese language teachers have rich expertise in teaching Chinese handwriting; another two researchers majoring in educational psychology, and mathematics education, respectively, have expertise in spatial cognition, and educational measurement and assessment; all these experts are native Chinese speakers and fluent in Chinese handwriting. An example handwriting sample that was previously scored using the CHLS was provided as a reference. With five additional products, the experts needed to apply the scale to these five and then fill out a feedback form (see details in Supplementary material) detailing their thoughts on the clarity of each criterion, the content breadth, and the degree to which they thought each criterion contributes to the construct of ‘Chinese handwriting legibility’. The experts were also asked to make any additional remarks on the CHLS.

### 2.2. Inter-rater reliability and construct validity

To assess the inter-rater reliability, two new raters were invited to independently score the products from 20 s grade students (randomly selected); this process was repeated for the products from 20 fourth and sixth grade students as well. Barnett et al. (2018) suggested that the narrower age band focusing on each scoring round could eliminate any discrepancies caused by age and render the discrimination between samples easier. The new raters were both 4th grade Chinese teachers with rich Chinese teaching experience throughout the whole elementary school; and they were trained by the first author to use the CHLS. Hence the raters’ standards in scaling can be unified to some extent. The first author scored all the handwriting products, and the total CHLS scores were divided into three categories in each grade: low, medium, and high (the mean minus/plus one standard deviation was set as the cut-off standard; Cascio et al., 1988; Barnett et al., 2018). The inter-rater reliability was calculated by applying the Cohen’s kappa coefficient between each new rater’s scores and the first author’s scores, and six inter-rater reliabilities were finally obtained.

To examine the construct validity, 50% of the participants’ products were randomly selected and subjected to a principal component analysis (PCA) to determine the number of components assessed by the CHLS. The remaining 50% of the participants’ products were subjected to confirmatory factor analysis (CFA) to further confirm the appropriateness of the factor structure explored using PCA. As boys’ handwriting is usually poorer than girls’ (e.g., Graham et al., 1998; Cui et al., 2012; Wicki et al., 2014), gender effects on each criterion and the total CHLS scores were also recorded to support the validity of the CHLS.

### 2.3. Internal consistency reliability and measurement invariance across grades

The internal consistency reliability of the entire sample was calculated using Cronbach’s alpha coefficient.

Measurement invariance (multi-group CFA), also with the entire sample, was assessed to examine the generalizability of the CHLS among the Grades 2, 4, and 6 students. A chi-square ratio (χ^2^/*df*) of ≤3 (Schermelleh-Engel et al., 2003), comparative fit index (CFI) of ≥0.95, Tucker–Lewis index (TLI) of ≥0.95, standardized root mean squared residual (SRMR) of ≤0.08, and root-mean-square error of approximation (RMSEA) of ≤0.06 (Hu and Bentler, 1999) were considered indicators of good model fits. For multi-group comparisons, the significance of ∆χ^2^ was used to judge the change in model fit between the compared models (Byrne, 2001).

### 2.4. Participants

A total of 684 students (367 boys, 317 girls) aged 8–12 years were recruited from a mainstream public primary school in Jingmen city in Hubei Province, China. Hubei Province is located in the central region of China, with a medium level of economic development. In the first three quarters of 2022 GDP ranking of provinces, Hubei ranked eighth with 3729.89 billion yuan among the 31 provinces; on this basis, located in the central district of Hubei Province, in its first three quarters of 2022 GDP ranking of cities, Jingmen city ranked seventh with 156.061 billion yuan among the thirteen cities. In this sense, with the middle economic development level, the Jingmen district should be representative of the average quality of basic education and academic level of students in central China. Due to personal limitations, with convenience sampling, only one mainstream public primary school in the urban area in Jingmen city was involved in the study, then 4, 5, and 4 classes from Grades 2, 4, and 6 were randomly chosen as the sample classes, respectively.

Of the 684 students, 209 were from Grade 2, 249 were from Grade 4, and 226 were from Grade 6 (Table 1). All participants are right-handed. According to the school records and teacher feedback, none of the students had developmental delays; dyslexia; neurological deficits; physical or mental challenges; or behavioral and emotional issues and sensory processing disorders.

**Table 1:** Number of boys and girls included in the study according to age.

Age (years)	Boys	Girls	Total
~8	115	94	209
~10	126	123	249
~12	126	100	226

### 2.5. Measures

#### 2.5.1. The SHARP handwriting task

As familiarity with and the complexity of the characters in the handwriting task can directly impact students’ evaluated performance, handwriting tasks should resemble what students regularly write at school but with varied difficulties (Li-Tsang et al., 2013). The template used by the SHARP evaluation for Chinese handwriting (Li-Tsang et al., 2022) was adopted in the present study; this template includes 90 simplified Chinese characters with font size 26, font type ‘KaiTi’, and triple-line spacing displayed in nine columns of 10 characters on an A4-sized paper. The template is designed on the one hand, based on how frequently the characters are used on a daily basis (Poon and Hong, 2003) – characters with low frequency were discarded from the selection so as to minimize the probability of students making errors because they had not learned that specific character before. On the other hand, the template covers all the six basic structures of Chinese characters (i.e., above–bottom, left–right, above–middle–bottom, left–middle–right, inside–outside, and independent) and 25 of the 30 basic stroke units (Law et al., 1998) to ensure the representativeness of the selected Chinese characters. Two 4th grade Chinese language teachers have also reviewed the template and confirmed its appropriateness for the Chinese primary school students in central China.

Contrary to the original experimental design requiring students to copy the characters on a digitized tablet without time limits, the present task required the students to copy the template on an A4-sized grid paper (top to bottom, left to right) as legibly as possible without compromising on speed within 4 min (but they did not need to copy the entire template). As demonstrated earlier, temporal characteristics reflect dynamics of handwriting process, signifying one critical dimension of handwriting evaluation; and the temporal pressure typically cause degradation of handwriting production (e.g., Gargot et al., 2020). Therefore, although this study does not involve the evaluation of handwriting dynamics, to ensure the potential comparability of the legibility results with prior work, time constraints were also adopted in this study. Additionally, requiring students to write with a certain speed can simulate the daily classroom setting, hence greatly reflecting their actual handwriting performance.

Within a time frame of 4 min, most of participants cannot finish the whole handwriting, and slow writers can copy at least first three lines. The similar research design can refer to existing handwriting research (e.g., Volman et al., 2006; Kaiser et al., 2009; Hellinckx et al., 2013; Van Hartingsveldt et al., 2015). The first author and the invited raters then assessed the handwriting legibility manually using the CHLS.

Moreover, to unify the assessment procedures, the environment set-up with the real-life handwriting context, and ergonomic factors such as the placement of handwriting materials, the use of writing accessories, lighting and noise, and writing posture were carefully monitored and controlled to ensure consistency among participants from different grades (Feder and Majnemer, 2007; Li-Tsang et al., 2013).

## 3. Data analysis

PCA and CFA were performed to determine and validate the factor structure of the CHLS, and the *t*-test was conducted to evaluate the gender differences in each criterion and the overall scores. Multi-group CFA was conducted to assess the measurement invariance of the CHLS. All data were analyzed using SPSS 26.0 and M*plus* 8.3.

## 4. Results

### 4.1. Content validity

After rating the five products using the CHLS, the experts provided independent feedback based on their expertise in Chinese handwriting and spatial cognition, practical experience working with students, and development and usage of other assessment instruments. The experts thought that most of the criteria were clearly presented, and further remarked that more scoring examples would be helpful to assist new raters in understanding the different criteria. Although comprehensive, some criteria, including h4, h7, and h8, were considered inappropriate to be applied to quantitative evaluation of Chinese handwriting legibility. h4 has rarely been emphasized in daily Chinese handwriting practice (although the lower grade students frequently use the square frame as reference in actual writing, teachers rarely emphasize the harms of out of grid/line; besides, in Chinese handwriting worksheet, there are not so many horizontal baselines as in alphabetic writing, hence the overshooting or undershooting the baseline in Chinese handwriting is negligible), hence the students performed with a certain randomness. h7 with superfluous/missing strokes generally may not impair the spacing/spatial relationships at the stroke or character level, nor the overall recognition of a specific character, considering that most Chinese characters are multi-stroke. As evidence, Graham et al. (2006) confirmed that in the copying task, h7 was typically used to characterize the construct of the motor program instead of the visual–spatial relationship or formation. Finally, h8 the extent of closure of stroke/radical varied depending on students’ handwriting styles and fonts. Especially in higher grades, with the handwriting fluency and styles develop, students pay little attention on the closure issue; even different writing tools can show different visual effects of closure. A closer inspection of the handwriting samples of the Grade 4 students based on these criteria suggesting that it is difficult to summarize regularity then determine standard on these rating supported the experts’ feedback. Moreover, the expert panel also provided some recommendations for the wording on the scoring sheet, such as revising the wording of h9 from ‘line formation’ to ‘line quality’, considering that h7–h10 collectively depict the stroke formation in the literature. Overall, the experts’ opinions were in favor of including the other seven criteria and their feedback clarified the pertinent descriptions and scoring guidelines.

### 4.2. Inter-rater reliability and construct validity

With h4, h7, and h8 excluded from the evaluation, the raw score of the seven-criteria CHLS ranges from 7 to 35. The score range and percent of each category for Grades 2, 4, and 6 are shown in Table 2. Take Grade 2 as an example, the ‘high’ category represents more than one standard deviation above the mean of the 209 participants (i.e., 25.77 plus 5.17 = 30.94 and rounded down to a score of 31).

**Table 2:** The mean, standard deviation, score range, and percent of each category for Grades 2, 4, and 6 of the seven-criteria CHLS.

	M	SD	Low	Medium	High
Grade 2	25.756	5.174	7 ≤ CHLS <21 (20%)	21 ≤ CHLS <31 (65.6%)	31 ≤ CHLS ≤35 (14.4%)
Grade 4	26.839	4.323	7 ≤ CHLS <23 (18%)	23 ≤ CHLS <31 (67%)	31 ≤ CHLS ≤35 (15%)
Grade 6	27.566	4.247	7 ≤ CHLS <23 (15.5%)	23 ≤ CHLS <32 (68.5%)	32 ≤ CHLS ≤35 (16%)

On this basis, the inter-rater reliability was good. For the Grade 2 students, the Cohen’s kappa coefficient between the first author and rater 1was 0.832 (*p* < 0.001), and between the first author and rater 2 was 0.916 (*p* < 0.001); for the Grade 4 students, the Cohen’s kappa coefficient between the first author and rater 1 was 0.706 (*p* < 0.001), and between the first author and rater 2 was 0.866 (*p* < 0.001); and for the Grade 6 students, the Cohen’s kappa coefficients between the first author and rater 1 was 0.752 (*p* < 0.001), and between the first author and rater 2 was 0.864 (*p* < 0.001).

Construct validity was assessed in two phases. In phase one, 50% of the students’ products were randomly selected and subjected to PCA (*N* = 341) using SPSS. The Kaiser–Meyer–Olkin (KMO) value in this study was 0.910; the results of Bartlett’s test of sphericity were significant (χ^2^ = 1266.795, *df* = 21; *p* < 0.001), indicating that it could be used for factor analysis. Furthermore, a single factor solution (with the eigenvalue being 4.364) was indicated through the examination of the screen plot and eigenvalues; this explained 62.336% of the variance observed. In phase two, the remaining participants’ products were subjected to CFA (*N* = 343), which was used to confirm the one-factor structure of Chinese handwriting legibility observed using PCA. The normality of the involved criteria was first checked to confirm that the precondition of maximum likelihood estimation was met. The absolute values of skewness and kurtosis should be <3 and < 8, respectively (Chen et al., 2005). In our study, the skewness ranged from −0.464 to −0.170 and the kurtosis ranged from −0.709 to −0.051, indicating that the criteria satisfied the standards of normality. On this basis, our CFA results indicated a satisfactory model fit (χ^2^ = 12.623, *df* = 11, χ^2^/*df* = 1.148, *p* = 0.3187; CFI = 0.999, TLI = 0.998, RMSEA = 0.021, 90% CI [0.000, 0.062], SRMR = 0.015). The factor loadings were all significant (*p* < 0.001), ranging from 0.598 to 0.905 (See Table 3).

**Table 3:** Factor loadings of the seven-criteria CHLS using CFA (*N* = 343).

CHLS criteria	Factor loadings
h1	0.905
h2	0.713
h3	0.708
h5	0.756
h6	0.598
h9	0.784
h10	0.704

In terms of the whole sample, mean scores for each criterion and the total CHLS for the boys and girls are displayed separately in Table 4. Significant gender differences were detected for each criterion and the total CHLS.

**Table 4:** Means, standard deviations, and *p* values of the boys’ and girls’ scores for each criterion and the total CHLS.

CHLS criteria	Boys (*N* = 367)		Girls (*N* = 317)		*p* value
	*M*	SD	*M*	SD	
h1	3.59	0.909	3.94	0.795	< 0.001
h2	3.75	0.885	4.04	0.762	< 0.001
h3	3.62	0.876	4.00	0.821	< 0.001
h5	3.68	0.792	3.92	0.763	< 0.001
h6	3.76	0.798	4.04	0.768	< 0.001
h9	3.59	0.898	3.85	0.829	< 0.001
h10	3.82	0.809	4.04	0.728	< 0.001
Total	25.81	4.812	27.83	4.153	< 0.001

### 4.3. Internal consistency reliability and measurement invariance across grades

The internal consistency of the CHLS was calculated using Cronbach’s alpha. The Cronbach’s alpha for all the measurement criteria was 0.902, indicating a high internal consistency. The item-total correlation varied between 0.742 to 0.858, and the alpha ranged from 0.877 to 0.894 when one of the criteria was deleted, indicating that deleting some criteria would not help improve the overall internal consistency (Table 5).

**Table 5:** The item-total correlation of the CHLS measurement criteria and the corresponding alpha if item deleted (*N* = 684).

CHLS criteria	Item-total correlation	Alpha if item deleted
h1	0.858	0.877
h2	0.802	0.886
h3	0.822	0.883
h5	0.760	0.891
h6	0.771	0.890
h9	0.793	0.888
h10	0.742	0.894

To assess the measurement invariance, the data normality of each grade was first tested. As shown in Table 6, all criteria for the three groups (Grade 2 [skewness: −0.345 to −0.060; kurtosis: −0.962 to −0.533], Grade 4 [skewness: −0.326 to 0.004; kurtosis: −1.004 to −0.256], and Grade 6 [skewness: −0.457 to −0.074; kurtosis: −0.728 to 0.268]) met the standards of normality for maximum likelihood estimation.

**Table 6:** Mean, factor loading, skewness, and kurtosis of the criteria in the three groups.

	Grade 2				Grade 4				Grade 6			
	M	FL	S	K	M	FL	S	K	M	FL	S	K
h1	3.59	0.881	−0.317	−0.534	3.77	0.856	−0.254	−0.535	3.89	0.883	−0.369	−0.473
h2	3.80	0.661	−0.331	−0.695	3.82	0.687	−0.326	−0.302	4.02	0.688	−0.457	−0.341
h3	3.70	0.717	−0.160	−0.856	3.76	0.739	0.070	−1.004	3.92	0.700	−0.416	−0.236
h5	3.62	0.721	−0.321	−0.533	3.85	0.743	−0.016	−0.590	3.88	0.708	−0.200	−0.356
h6	3.69	0.657	−0.060	−0.937	3.91	0.583	−0.159	−0.256	4.06	0.653	−0.231	−0.594
h9	3.56	0.756	−0.186	−0.962	3.74	0.760	0.004	−0.743	3.82	0.806	−0.074	−0.728
h10	3.80	0.692	−0.345	−0.834	3.99	0.719	−0.045	−0.923	3.97	0.703	−0.202	0.268

FL, factor loading; S, skewness; K, kurtosis.

Then, separate CFAs were conducted for the Grades 2, 4, and 6, respectively. The results showed that the model fits were satisfactory (Table 7), and the factor loadings were all significant and almost >0.60 (Table 6), indicating that the one-factor structure was appropriate for all three groups.

**Table 7:** Model fit statistics for CFAs in the three groups.

Groups	*N*	χ^2^ (*df*)	χ^2^/*df*	CFI	TLI	RMSEA	SRMR
Grade 2	209	14.343 (11)	1.304	0.996	0.992	0.038	0.019
Grade 4	249	12.624 (11)	1.148	0.998	0.996	0.024	0.018
Grade 6	226	16.106 (11)	1.464	0.994	0.989	0.045	0.018

CFI, comparative fit index; TLI, Tucker–Lewis index; RMSEA, root-mean-square error of approximation; SRMR, standardized root mean squared residual. The same below.

On this basis, to detect the cross-grade generalizability of the CHLS, three nested models were compared. In the configural model, all parameters were freely estimated. The metric model fixed the factor loadings among all groups. In the scalar model, both factor loadings and intercepts were constrained.

As shown in Table 8, the fits of the configural and metric models were not significantly different (*p* = 0.089), indicating that the factor loadings were invariant, and the metric invariance was established among the three groups. Scalar invariance was examined by further fixing the intercepts among the groups. A significant change was identified (*p* < 0.05; ∆CFI = 0.011 > 0.01, ∆RMSEA = 0.017 > 0.005; see Chen, 2007; Putnick and Bornstein, 2016), indicating that the scalar invariance of the CHLS was not supported across the three groups.

**Table 8:** Measurement invariance results of the CHLS for Grades 2, 4, and 6.

	χ^2^ (*df*)	CFI	TLI	RMSEA	SRMR	∆χ^2^ (∆*df*)	∆CFI	∆RMSEA
Configural model	42.340 (33)	0.996	0.993	0.035 [0.000, 0.063]	0.018			
Metric model	61.305 (45)	0.994	0.991	0.040 [0.000, 0.063]	0.068	18.965 (12) *p* = 0.089	−0.002	0.005
Scalar model	99.959 (57)	0.983	0.982	0.057 [0.038, 0.076]	0.072	38.654 (12) *p* < 0.000**	0.011	0.017

**p < 0.01, two-tailed. The same below.

To identify the specific reason for this non-invariance, we separately assessed the measurement (especially scalar) invariance between two of the three groups. The results (Tables 9–11) showed that scalar invariance was supported between Grades 4 and 6 and between Grades 2 and 6 but not between the Grades 2 and 4. Further, focusing on the partial scalar invariance between Grades 2 and 4 (Table 9), by sequentially releasing criterion intercept constraints and retesting the model, it was found that when h5 and h6 were freely estimated, the partial scalar invariance was supported, indicating the differences of h5 and h6 between Grades 2 and 4.

**Table 9:** Measurement invariance results of CHLS for the Grades 2 and 4.

	χ^2^ (*df*)	CFI	TLI	RMSEA	SRMR	∆χ^2^ (∆*df*)	∆CFI	∆RMSEA
Configural model	23.015 (22)	0.999	0.999	0.014 [0.000, 0.058]	0.017			
Metric model	32.761 (28)	0.997	0.996	0.027 [0.000, 0.060]	0.053	9.746 (6) *p* = 0.136	0.002	0.013
Scalar model	51.449 (34)	0.990	0.988	0.047 [0.016, 0.072]	0.070	18.689 (6) *p* < 0.005**	0.007	0.02
Partial scalar model (h5, h6 were freed)	37.985 (32)	0.997	0.995	0.029 [0.000, 0.059]	0.056	5.224 (4) *p* = 0.265	0.000	0.002

**Table 10:** Measurement invariance results of CHLS for the Grades 4 and 6.

	χ^2^ (*df*)	CFI	TLI	RMSEA	SRMR	∆χ^2^ (∆*df*)	∆CFI	∆RMSEA
Configural model	28.730 (22)	0.996	0.993	0.036 [0.000, 0.069]	0.018			
Metric model	36.911 (28)	0.995	0.992	0.037 [0.000, 0.066]	0.057	8.181 (6) *p* = 0.225	−0.001	0.001
Scalar model	47.761 (34)	0.992	0.990	0.041 [0.000, 0.067]	0.052	10.851 (6) *p* = 0.093	−0.003	−0.002

**Table 11:** Measurement invariance results of CHLS for the Grades 2 and 6.

	χ^2^ (*df*)	CFI	TLI	RMSEA	SRMR	∆χ^2^ (∆*df*)	∆CFI	∆RMSEA
Configural model	26.497 (22)	0.997	0.995	0.031 [0.000, 0.067]	0.017			
Metric model	34.945 (28)	0.996	0.994	0.034 [0.000, 0.066]	0.058	8.447 (6) *p* = 0.207	−0.001	0.003
Scalar model	46.123 (34)	0.993	0.991	0.040 [0.000, 0.068]	0.064	11.178 (6) *p* = 0.083	−0.003	−0.006

On this basis, ANOVA-test (and *post hoc* multiple comparison) among the means of each criterion for the Grades 2, 4, and 6 was conducted to further compare students’ performance in each criterion. The results showed that when students progressed from Grade 2 to Grade 4, except for h2 and h3, significant performance improvements were noted for other criteria; whereas when students progressed from Grade 4 to Grade 6, only h2, h3, and h6 presented significant improvements; overall, students significantly progressed on each criterion from Grade 2 to Grade 6 (Table 12).

**Table 12:** Means, standard deviations, and results of the ANOVA-test (and *post hoc* multiple comparison) for the Grades 2, 4, and 6 for each criterion of the CHLS.

CHLS	Grade 2 (*N* = 209)		Grade 4 (*N* = 249)		Grade 6 (*N* = 226)		F	*p* value	*p* value (Grade 2 vs. 4)	*p* value (Grade 4 vs. 6)	*p* value (Grade 2 vs. 6)
	M	SD	M	SD	*M*	*SD*					
h1	3.59	0.916	3.77	0.848	3.89	0.844	6.358	0.002**	0.033*	0.126	0.000**
h2	3.80	0.912	3.82	0.804	4.02	0.802	4.681	0.010*	0.804	0.010*	0.007**
h3	3.70	0.930	3.76	0.868	3.92	0.804	3.656	0.026*	0.428	0.050*	0.009**
h5	3.62	0.870	3.85	0.728	3.88	0.745	7.581	0.001**	0.001**	0.640	0.000**
h6	3.69	0.932	3.91	0.698	4.06	0.719	12.111	0.000**	0.003**	0.038*	0.000**
h9	3.56	0.980	3.74	0.828	3.82	0.808	5.349	0.005**	0.025*	0.294	0.001**
h10	3.80	0.960	3.99	0.727	3.97	0.625	4.010	0.019*	0.010*	0.839	0.020*

*p < 0.05, **p < 0.01, two-tailed.

## 5. Discussion

The logographic nature and visual–spatial properties of Chinese characters determine the uniqueness and complexity of Chinese handwriting, and its close association with spatial cognition (Chinese-character writing’s psycho-geometric theory; Kao, 2000). Numerous cross-cultural comparative studies support this proposition. Combined with the spatial-oriented characteristics of the legibility dimension, the spatial-related nature of Chinese handwriting legibility is further strengthened. In other words, spatial thinking is implicit in legible Chinese character writing, and there is an implicit association between Chinese handwriting legibility and spatial cognition. In this regard, evaluation and diagnosis of Chinese handwriting legibility taking into account a detailed spatial analysis at the analytic and holistic levels, is of great significance for student development and teacher instruction, and the CHLS in the present study was developed in this context.

The seven criteria of the CHLS are supported by the literature and the experts’ reviews. Spacing/spatial relationships between strokes/radicals are evaluated based on the extent to which the components are positioned correctly; examples of errors include but are not limited to overlaps or writing too far apart, collisions and adhesions of components, and dislocation of components. This criterion is an analytic-level measure relative to the spaces between characters, which assess whether and how the characters are evenly separated in the whole script. Alignment focuses on the overall horizontal and vertical layout of the characters. Size uniformity is evaluated at the analytic (focusing on specific strokes/radicals within a character) and holistic (focusing on characters in the whole text) levels. Line quality is another crucial criterion, with errors often originating from poor line formation (e.g., if the curves are angular or straight lines are wavy). Finally, writing in the appropriate direction also facilitates readability (e.g., Parush et al., 2010; Klein et al., 2011). This include no deviations in the orientation of specific strokes, and the character is oriented vertically relative to the horizontal line. These criteria concur well with and complement the claim of Li-Tsang et al. (2022) that the location, proportion, size, and direction of the strokes are all vital for legible Chinese handwriting.

Inter-rater consistency is a critical indicator in weighing the reliability of the CHLS in its application to different raters. According to Barnett et al. (2018)’s method in examining the inter-rater reliability of handwriting scales, the participants’ total scores of Chinese handwriting legibility performance of each grade were categorized into the low, medium, and high groups. The inter-rater reliabilities based on these classifications were good but could be further improved. An in-depth analysis uncovered that this was primarily due to the moderate agreement between raters on the ‘direction’ criterion. Subsequent discussions with the raters indicated that they were somewhat uncertain about assessing strokes/radicals with deviated directions and samples with personalized handwriting styles/fonts. This finding highlights the necessity to further refine and clarify the instructions and provide more examples for future raters.

As suggested by Barnett et al. (2007), a cut-off score is necessary for a test to identify those with poor performance and even difficulties. To achieve this goal, we divided the CHLS total scores into low, medium, and high categories in each grade so as to more accurately identify the sample with poor Chinese handwriting legibility. With a normal distribution, more than 15% of the students in each grade has been identified with potential handwriting difficulties, which aligns with the frequency reported in existing literature (e.g., Feder and Majnemer, 2007). In this sense, the CHLS might be an appropriate screening tool concerning legibility problems; at present we would recommend these levels (Table 2) to identify children with poor Chinese handwriting legibility, and likely in need of instruction support.

The construct validity depicts how well a scale measures the construct it is intended for. The CHLS was developed to assess the detailed spatial characteristics of Chinese handwriting legibility at both the analytical and holistic level, and PCA of the seven criteria uncovered a one-factor solution explaining a large proportion of the variance. This result supported the significance of these criteria in constructing the overall legibility dimension. Furthermore, the factor loadings of the CHLS criteria were relatively high for h1, h5, and h9; whereas were lower for h2 and h6. The size tendency of factor loadings may reflect on the one hand, the prominent role of analytic criteria in the legibility dimension, and on the other hand, the differences between the analytic versus holistic criteria. The CFA results further supported this one-factor structure. Besides, the significant gender differences observed in this study, that is, boys generally performed lower in legibility than girls, is consistent with existing research (e.g., Graham et al., 1998; Cui et al., 2012; Wicki et al., 2014), hence also supplements the validity of the CHLS to some extent.

Combined with the high internal consistency reliability showing that the seven criteria were closely related with the total score, all these reliability and validity results suggest that the CHLS developed in this study is appropriate for measuring the Chinese handwriting legibility performance of Chinese primary students in central China.

On this basis, measurement invariance of the CHLS was further assessed for students across Grades 2, 4, and 6. The results confirmed the metric invariance of the CHLS across the three grades, suggesting that the factor structure of the CHLS, and relative contribution (i.e., the factor loading) of each criterion to the latent construct were consistent for students of these three grades. This invariance, to some extent, supported the generalizability of the CHLS in the Chinese primary school context in central China. Further measurement invariance tests between two of the three grades revealed the scalar invariance between Grades 4 and 6 and between Grades 2 and 6, but significant intercept variations (i.e., h5 and h6) between the Grades 2 and 4. As Cheung and Rensvold (2002) claimed, cross-group differences detected in multi-group CFA may be valuable for understanding ‘how different groups view the world’ (p. 252). In other words, different groups may hold distinct attitudes, perceptions, or ratings on the criteria concerned. In the context of this study, the cross-group differences in terms of h5 and h6 of the CHLS may reflect the developmental specificity of students’ Chinese handwriting legibility in size across lower grades. Although to the researchers’ knowledge, there are no existing research in handwriting development explicitly confirmed this finding, children with poor handwriting legibility or dysgraphia, or namely low handwriting ability, usually present the greater variability of stroke/radical/character size (e.g., Hamstra-Bletz and Blöte, 1993; Marr et al., 2001; Volman et al., 2006) support this finding to some extent. Furthermore, a thorough comparison of each criterion across grades can shed more light on the developmental specificity of students’ Chinese handwriting legibility. Specifically, the *post hoc* multiple comparison results between Grades 2 and 4 further revealed that students’ Chinese handwriting legibility significantly progressed at the analytic level from Grade 2 to Grade 4; whereas there were no *corresponding* significant differences between Grade 4 and Grade 6, i.e., the relevant skills became relatively mature and automatic after Grade 4. This change may be closely related to students’ Chinese character learning experiences. In the early grades, due to students’ relatively limited capacity, handwriting instruction, learning and assessment usually focus more on the precise spatial analysis and processing of specific strokes, radicals, or other subcomponents, which may contribute to their significant progression in legibility performance at the analytic level. However, when students enter the senior grades, with their accumulation of Chinese handwriting experience, formal handwriting instruction in class reduces, and teachers focus more on the content and fluency of students’ work rather than their analytical legibility (Lam et al., 2011). Hence students’ Chinese handwriting legibility performance may reach a plateau at this stage. Noteworthy, although the time invested for formal handwriting instruction reduces in Grades 4–6, as Lee et al. (2016) claimed, Chinese students learn new Chinese characters throughout elementary school, and they continuously adhere to all orthographic rules and are consistently reminded to assure the quality of their handwriting output. These constant handwriting requirements might greatly contribute to students’ satisfactory legibility in the senior grades, as observed in the present study. Indeed, there were significant improvements in h2, h3 and h6 behind the scalar invariance from Grade 4 to Grade 6. This finding is valuable for it reflects the legibility improvement at the holistic level. Similarly, despite the scalar invariance between Grade 2 and Grade 6, there were significant improvements in handwriting legibility performance at both the analytic and holistic levels. All these changes signify that there exists developmental specificity in these students’ Chinese handwriting legibility performance: among the different criteria, handwriting size presents greater variability across the lower grades; and the early ability improvement is more reflected at the analytic level, whereas in the senior grades it centers around the holistic level.

The complex and dynamic nature of handwriting activities may also contribute to these students’ developmental specificity in Chinese handwriting legibility performance. According to Feder and Majnemer (2007), handwriting is a complex human activity interweaving perceptual motor, cognition, and tactile and kinesthetic sensitivities. Kao (2000) further refined that Chinese handwriting involves the dynamic integration of the writer’s perception, cognition, and motor components. These definitions imply that there involves inevitably differential skill mobilization behind students’ handwriting progression, which can to some extent act as the theoretical basis favoring the transfer of spatial relationship emphasis from the analytic level to the holistic level in handwriting with students’ growth in this study. More empirical research is demanded in the future to examine the plausibility of this explanation.

Lastly, it’s worthy to note that despite the present study highlights spatial thinking, or specifically spatial cognitive processing needs behind the seven criteria of Chinese handwriting legibility, it is undeniable that other criteria/factors also make a difference in handwriting skill development. For example, as Li-Tsang et al. (2013) demonstrated, despite being undetectable in written production, reversal of strokes/radicals and sequence errors could also be the possible reasons causing handwriting difficulties. Tseng and Murray (1994) indicated that learning disabilities or behaviors linked to attention disorders may impair the acquisition and maintenance of handwriting proficiency. Besides, through a review of the development of the aural, oral, reading, and writing systems in school children, Berninger (2000) concluded that these communication systems are interrelated with changes in one influencing the development of the others. In this sense, the present study is still the initial work, and a more refined research design with those factors controlled is needed in future work.

## 6. Limitations and implications

It is essential to keep in mind the limitations of this initial work, and the relevant results should be interpreted with caution. First, only Chinese primary school students from central China were included in this study, making the presented CHLS constrained in its application in the broad Chinese context. Future research should expand the sampling area to examine and improve the instrument’s generalization. Second, although the relative importance of different spatial criteria in the Chinese handwriting legibility assessment is reflected, the standard-setting of the CHLS is based on simplified Chinese characters, a specific type of logographic script, and its applicability to other graphic scripts (e.g., traditional Chinese characters, McBride, 2016; Japanese kanji, Sakamoto and Spiers, 2014) remains uninvestigated. Given the possible differences in the representational characteristics of different scripts, we remain cautious in extending our findings to other scripts. Third, as with any individual research, the reliability and validity examinations conducted in this study were limited, and further work is still needed to supplement the CHLS’s psychometric properties. For example, based on the expert panel and authors’ qualitative evaluation, providing more quantitative evidence on the criteria inclusion/exclusion would afford more rigor to the content validity. This study would also be more comprehensive if children with specified disorders (e.g., Attention Deficit Hyperactive Disorders (ADHD), Developmental Coordination Disorder (DCD), which are commonly known to be connected with handwriting problems; e.g., Mayes and Calhoun, 2006; Barnett et al., 2018) and typical development could be incorporated to examine its discriminant validity. Besides, comparing the CHLS to other measures of handwriting legibility, such as computerized measures and eyeballing analysis by supervising teachers or parents of the relevant criteria, would provide information about the convergent validity of the CHLS (e.g., Li-Tsang et al., 2013). Fourth, the sensitivity of the CHLS to evaluate changes in handwriting legibility performance related to implementing a particular intervention or support remains unresolved. The relevant empirical evidence is needed to further support the application of the CHLS in clinical or instructional contexts. Fifth, although the handwriting produced in the required time is recorded alongside the CHLS instructions and can indicate the production rate, it is not the focus of this study, and a more rigorous and formal evaluation of handwriting speed is recommended in any subsequent assessment. Last but not the least, relying only on school records and teachers’ feedback, but no rigorous and specific tests were conducted to identify and exclude ADHD and other implicit disorders from the sample, might potentially introduce some confounding effects on the present students’ poor performance in Chinese handwriting legibility. Future work should strictly scrutinize the sample so as to avoid this phenomenon.

Notwithstanding these limitations, the CHLS is the first handwriting evaluation scale focusing on the detailed spatial aspects of the legibility dimension in the Chinese primary school context. Theoretically, the establishment of the CHLS can extend the Chinese handwriting theory and shed light on a deeper understanding of Chinese characters and Chinese handwriting, which may lay the foundation for further uncovering the underlying mechanism of their contribution to and linkage with spatial cognition. Practically, the CHLS may be a useful tool that can be used to evaluate the Chinese handwriting legibility performance of students with and without handwriting difficulties. It has potential to favor analyzing and understanding the characteristics of students with handwriting difficulties from the perspective of spatial cognition, thus affording teachers and clinicians guidance on handwriting support and intervention accordingly.

## 7. Conclusion

Given the close relationship between Chinese handwriting and spatial cognition, and the spatial-oriented characteristics of the legibility dimension, we developed and validated a tailored assessment scale for Chinese handwriting legibility based on analyzing its spatial characteristics at both the analytic and holistic levels. With data from 684 Grades 2, 4, and 6 Chinese primary school students from central China, the CHLS showed good inter-rater reliability, and satisfactory construct validity and internal consistency reliability. The results of the measurement invariance confirmed the generalizability (in terms of factor structures and loadings) of the CHLS in these primary school students in various grades. Our results also highlight the developmental specificity of these students’ Chinese handwriting legibility performance with their growth.

### What this paper adds

This paper demonstrates the close relationship between Chinese handwriting and spatial cognition and claims spatial-oriented characteristics of the legibility dimension; in this context, we developed and validated the Chinese Handwriting Legibility Scale (CHLS), a new tool focusing on a detailed analysis of spatial characteristics of Chinese handwriting legibility performance. Based on previous research on (Chinese) handwriting legibility and relevant scales and tools, the CHLS is established with spatial-related criteria at both the analytic and holistic levels. With 684 Chinese primary school students from Grades 2, 4, and 6 in central China as participants, the CHLS presented good inter-rater reliability, satisfactory construct validity and internal consistency reliability. This study also furthers the field with measurement invariance examination. The result, on the one hand, confirms the generalizability (in terms of factor structures and loadings) of the CHLS in Chinese primary school students in central China in various grades, on the other hand, highlights the developmental specificity of these students’ Chinese handwriting legibility performance with their growth.

## Data availability statement

The datasets presented in this article are not readily available because this dataset is one part of the first author's thesis study and cannot be shared because it involves confidential information of participants. Requests to access the datasets should be directed to HL, luhong2018@connect.hku.hk.

## Ethics statement

The students’ parents/legal guardians and schoolteachers and the school principal were informed of the study purpose and provided informed consents supporting the children’s participation in the study. The students signed the assent form before the tests were administered. This study was ethically approved by the Human Research Ethics Committee of the University of Hong Kong.

## Author contributions

HL and FL contributed to the conception and design of the study. HL organized the database and wrote the first draft of the manuscript. HL and XC performed the statistical analysis. All authors contributed to manuscript revision, read, and approved the submitted version.

## Funding

Publication made possible in part by support from the HKU Libraries Open Acccess Author Fund sponsered by the HKU libraries.

## Conflict of interest

The authors declare that the research was conducted in the absence of any commercial or financial relationships that could be construed as a potential conflict of interest.

## Publisher’s note

All claims expressed in this article are solely those of the authors and do not necessarily represent those of their affiliated organizations, or those of the publisher, the editors and the reviewers. Any product that may be evaluated in this article, or claim that may be made by its manufacturer, is not guaranteed or endorsed by the publisher.
