# Supplementary material for: Reliability, validity, and measurement invariance of a Chinese handwriting legibility scale among primary students in central China
Source: Front Psychol. 2023 Jul 28;14:1050894. doi: 10.3389/fpsyg.2023.1050894 (PMC10422027; doi:10.3389/fpsyg.2023.1050894)
Supplement: Supplementary file 1 [file Data_Sheet_1.PDF]

Supplementary material:

Handwriting evaluation in general:

Name of the instrument	Age	Scoring method	Script	Criteria	Application
Chinese Handwriting Evaluation Form (CHEF; Chang & Yu, 2012)	Grades 1-2	A 5-point Likert scale (1: never matching to 5: always matching)	Chinese characters	1. Construction: size, spacing, and alignment of characters and components<br>2. Accuracy: the malformation of characters, i.e., incorrect figuration of components, adding or missing strokes<br>3. Speed<br>4. Pencil grasp<br>5. Directionality	Shin et al., 2018
Chinese Handwriting Evaluation Questionnaire (CHEQ; Chang & Yu, 2005)	Grade 2	Unspecified	Chinese characters	1. Legibility<br>2. Accuracy<br>3. Speed<br>4. Pencil grip<br>5. Gross movement<br>6. Attitude	Chang & Yu, 2005
The Tseng Handwriting Problem Checklist (THPC; Tseng, 1993)	Grades 1-4	A 4-point scale, ranging from 1 = always to 4 = seldom	Chinese characters	1. Construction<br>2. Accuracy<br>[1&2 reflect measures of legibility]<br>3. Behavior<br>4. Sequencing<br>5. Motor<br>6. Directionality	Shen et al., 2012

The Handwriting Legibility Scale (HLS; Barnett et al., 2013, 2018)	Children aged 8-14	A scale from 1-5, with 1 representing the best performance	Alphabet	1. Global legibility (overall readability of the text on first reading)<br>2. Layout on the page<br>3. Letter formation<br>4. Effort to read the script<br>5. Alterations to writing (attempts made to rectify written work)	Prunty et al., 2016
The Handwriting Proficiency Screening Questionnaire (HPSQ; Rosenblum, 2008)	Grades 2-8	A 5-point scale (0 refers to never and 4 refers to always)	Alphabet	1. Unreadable handwriting<br>2. Unsuccessful in reading his/her own handwriting<br>3. A lack of time to copy<br>4. Often erases<br>5. Does not want to write<br>6. Does not do homework<br>7. Complains about pain<br>8. Tired while writing<br>9. Needs to look often when copying<br>10. Not satisfied with his/her handwriting<br>Among them, items 3 through 9 represents the factor of 'performance time and well-being', whereas items 1, 2, and 10 denotes 'legibility' factor.	Rosenblum, 2008
Detailed Assessment of Speed of Handwriting (DASH test; Barnett et al., 2007)	Aged 9-16	To calculate the writing speed, the total of legible words divided by the	Alphabet	The quality and speed of writing under different conditions including copying and generating text.	Barnett et al., 2007

		time of the test is counted.<br><br>The legible letters are counted and in the correct sequence.<br><br>In task "graphic speed", the speed is counted by the correct number of "X" inside the circles.			

Computerized handwriting evaluation:

Name of the instrument	Age	Scoring method	Script	Criteria	Application
The Chinese Handwriting Assessment Tool (CHAT; Li-Tsang et al., 2011, 2013)	Grades 1-6	Computerized real-time recording	Chinese characters	1. Accuracy: no. of correctly written characters/90 characters<br>2. Speed: no. of characters copied per min, pause time to on-paper time ratio<br>3. Pressure: mean pen pressure and variability<br>4. No. of characters exceeded grid<br>5. Average size of characters (mm)	Cheng-Lai et al., 2013; Lam et al., 2011

				6. Variability (SD) of character size (mm)	
The Smart Handwriting Analysis and Recognition Platform (SHARP; Li-Tsang et al., 2022)	Grades 1-6	Computerized real-time recording	Chinese characters	1. Handwriting process (ground time, air time, air/ground time ratio, speed, SD of writing time per character, pen pressure, SD of pressure);<br>2. Handwriting product (out of grid, size, SD of size, identified words, wrong stroke, additional stroke, missing stroke, concatenated stroke, reverse stroke, wrong stroke sequence)	Li-Tsang et al., 2022
The Computerized Legibility Assessment (CLA; Lee et al., 2016)	Grades 1-6	Computerized real-time recording	Chinese characters	1. Length of every stroke<br>2. Orientation of every stroke<br>3. Placement of every stroke<br>4. Task completion time<br>5. Stroke velocity<br>6. Stroke force<br>7. Pause time per stroke	Lee et al., 2016
Computerized apparatus and handwriting task (Shen et al., 2012)	Grades 1-4	Computerized real-time recording	Chinese characters	The positions, sequence of strokes, and pressure of writing:<br>1. Total writing time<br>2. Total in-air time<br>3. Total on-paper time, which is the length of time the pen touches paper<br>4. In-air trajectory<br>5. Speed (cm/s): the length of distance when pen touches paper 6. Axial pen pressure<br>7. Average character width<br>8. Average character height	Shen et al., 2012

Name Writing Task (Taverna et al., 2020)	Grade 1	Computerized real-time recording	Alphabet	1. Speed/Frequency of strokes: frequency of upward and downward movements in 1 s<br>2. Stroke pressure<br>3. Automaticity: no. of inversion of velocity	Taverna et al., 2020
Handwriting Tasks (Wicki et al., 2014)	Grade 4	Computerized real-time recording	(Swiss-German) Alphabet	1. Speed/Stroke frequency: no. of upward and downward movements in 1 s<br>2. Stroke pressure<br>3. Automaticity: no. of inversion of velocity	Wicki et al., 2014
The Computerized Penmanship Evaluation Tool (POET; Rosenblum et al., 2003)	Children aged 8-9	Computerized real-time recording	Alphabet	1. Spatial measure: the total path length on the paper of all the characters written in the paragraph<br>2. Temporal measure: the time taken to write each segment, the total time taken to complete the entire paragraph, on-paper time, and in-air time<br>3. Pressure measure: the mean pressure applied to the paper	Mekyska et al., 2017
A digital diagnostic tool (Pagliarini et al., 2017)	Grade 1-5	Computerized real-time recording	Alphabet	Kinematics and trajectory of handwriting	Pagliarini et al., 2017
A digital diagnostic tool (Mekyska et al., 2017)	Grade 3 (aged 8 and 9)	Computerized real-time recording	Alphabet	1. Kinematic Measures (speed, velocity, acceleration, jerk, normalized jerk, height, orientation, duration, and length)<br>2. Nonlinear dynamic features<br>3. Other Features	Mekyska et al., 2017
A digital diagnostic tool (Asselborn et al., 2018)	Children	Computerized real-time recording	Alphabet (Latin)	1. The geometrical aspect of handwriting,<br>2. The use of pressure, tilt, and kinematics.	Asselborn et al., 2018

A digital diagnostic tool (Gargot et al., 2020)	Children	Computerized real-time recording	Alphabet	1. Static characteristics (purely geometrical characteristics of the handwriting text: space between words, SD of handwriting density, and median of power spectral of tremor frequencies);<br>2. Kinematic features (dynamics of the handwriting process: median of power spectral of speed frequencies, distance to mean of speed frequencies, in-air-time ratio);<br>3. Pressure features (pressure measured between the pen tip and the tablet surface: average pressure, mean speed of pressure change, SD of speed of pressure change);<br>4. Tile features (tilt between the pen and the surface of the tablet: distance to mean of tilt-x frequencies, the bandwidth of speed of tilt-x frequencies, median of power spectral of tilt-y frequencies).	Gargot et al., 2020

Handwriting legibility evaluation:

Name of the instrument	Age	Scoring method	Script	Criteria	Application
Scale (authors)	Adaptive ages	Rating method	Script type	Criteria and indicators	Research application
The Persian Handwriting Assessment Tool (PHAT; Havaei et al., 2017)	Grades 2-3	A 5-point scale, ranging from very poor to very good	(Tajik) alphabet	1. Formation<br>2. Space<br>3. Alignment<br>4. Size<br>5. Text slant	Farhangnia et al., 2020; Seyyedrezaei et al., 2021

The Minnesota Handwriting Assessment (MHA, Reisman, 2004)	Children aged 7-12	One point for each correct letter following specified criteria	Alphabet	1. Legibility<br>2. Spacing<br>3. Alignment<br>4. Size<br>5. Form	Bo et al., 2014;<br>Bumin & Kavak, 2010
The Scale of Children's Readiness In PrinTing (SCRIPT; Weil & Cunningham Amundson, 1994)	Kindergarten	One point for each correct letter following specified criteria	Alphabet	Correct:<br>1. The letter is recognizable and legible.<br>2. All parts of the letter are complete, e.g., i must be dotted; f and t must be crossed; m, u, r, d and similar letters (h, b, p, n) all contain the straight line and not just curves (d looking like a reversed 6 is counted as incorrect); g, q, and/must have curves on descenders and ascenders; n, m, and w need to be proportionate to 1/4 in. of the body of the letter's length.<br>3. The letter is proportionate in size (parts, body).<br>Incorrect:<br>1. The letter is reversed (typically b, d, p. q).<br>2. The letter is rotated more than 45° from proper orientation.<br>3 An uppercase letter is substituted for a lowercase letter or vice versa.<br>4. The letter contains additional parts (e.g., an m contains more than two humps).<br>5. The letter is not printed within the box below	Daly et al., 2003;<br>Desai & Rege, 2004

				the model letter.<br>6. The letter is in two or more distinct parts. A break in a line of less than 1/16 in. is permitted.<br>7. In the letters a, b, d, m, n, p, q, and r, the straight line extends more than 1/4 in below or above the body of the letter or is not proportionate.	
The Hebrew Handwriting Evaluation (HHE; Erez et al., 1996)	Grades 1-4	A scale from 1-4, with 1 representing the best performance	Alphabet (Hebrew)	1. Legibility: global legibility, letter formation [e.g., closure, reversals]<br>2. Spatial arrangement/organization [e.g., consistency, spacing--absence of gaps or overlaps of letters/words, letter size, alignment, margins, and straight lines]<br>3. Direction: writing letters in the appropriate direction<br>4. Speed<br>5. Posture [e.g., positioning of the paper and stability of head and neck]	Gilboa et al., 2010, 2014; Parush et al., 2010; Preminger et al., 2004; Rosenblum, 2008; Yochman & Parush, 1998
The Handwriting Evaluation Scale (Malloy-Miller, 1985)	Grade1 1-6	Percentage of errors versus total number of letters printed or written	Alphabet	1. Spacing within words (overlapping letters or letters too far apart)<br>2. Spacing between words (too little, too much or no space between words)<br>3. Size of letters within words (whole or part of letter is too big or too small)<br>4. Size between words (some words are small and some words are big)	Malloy-Miller, 1985

				5. Baseline orientation (letters overshoot or undershoot the baseline)<br>6. Closure (improper closure of letter parts)<br>7. Line quality (curves are angular or straight lines are wavy	
The Concise Assessment Scale for Children's Handwriting (BHK; Hamstra-Bletz et al., 1987);<br><br>The revised version: the Dutch tool Systematic Screening of Handwriting (Dutch: 'Systematische Opsporing van Schrijfmotorische Stoornissen', i.e., SOS; Van Waelvelde et al., 2009, 2012)	Grades 2-3	An ordinal scale from 0 to 5, a high score indicating deviance	Alphabet	1. Letter size too large for the child's age<br>2. Left margin widening<br>3. Poor word alignment<br>4. Insufficient word spacing<br>5. Acute turns in connecting letters<br>6. Irregularities in joining letters<br>7. Collision of letters<br>8. Inconsistent letter size<br>9. Incorrect relative height<br>10. Odd letters<br>11. Ambiguous letter forms<br>12. Correction of letter forms<br>13. Unsteady writing trace	Duiser et al., 2014; Hellinckx et al., 2013; Kaiser et al., 2009; Overvelde & Hulstijn, et al., 2011; van Hartingsveldt et al., 2015; Volman et al., 2006
The Tseng Handwriting Problem Checklist (Tseng, 1993)	Grades 4-5	A 3-point Likert scale (0 = most legible, 2 = least legible)	Chinese characters	1. Square configuration (i.e., out of grid)<br>2. Number of strokes (i.e., superfluous/missing strokes)<br>3. Spatial relationship (i.e., incorrect position of components, poor alignment of characters)	Linda et al., 2014

				4. Spacing and size (i.e., disproportional spacing and size between components of a character)<br>5. Word formation (i.e., malformation of components)	
The Evaluation Tool of Children's Handwriting-Manuscript (ETCH-M; Amundson, 1995)	Grades 1-2	Illegible words/letters/numerals are counted and converted to a word/letter/numeral percentage; total ETCH-M word, letter, numeral legibility scores are obtained and expressed as total legibility percentages.	Alphabet (and numerals)	1. Letter formation<br>2. Size<br>3. Horizontal alignment<br>4. Spacing<br>5. Letter case<br>6. Speed	Amundson, 1995
Self-developed handwriting evaluation 1 (Klein et al., 2011)	Grades 3-6	The number of errors made	Alphabet	1. Letter formation<br>2. Size<br>3. Spacing<br>4. Alignment<br>5. Slant<br>6. Order<br>7. Reversals<br>8. Omissions<br>9. Insertions<br>10. Tremulous/jerky lines<br>11. Margin widening and/or narrowing	Klein et al., 2011

				12. Pressure	
Self-developed handwriting evaluation 2 (Maeland, 1992)	Grade 4	A 7-point scale	Alphabet	1. Accuracy of letter formation<br>2. Uniformity of letter size<br>3. Uniformity of letter slope<br>4. Spacing between letters and words<br>5. Alignment of the lines of writing	Maeland, 1992
Self-developed handwriting evaluation 3 (Tseng, 1994)	Grades 3-5	A 7-point scale	Chinese characters	Global legibility sorting	Tseng, 1994
Self-developed handwriting evaluation 4 (Jameel et al., 2017)	Grades 4-5	A 5-point scale, with 1 to 5 being given to poor and excellent legible handwriting	Alphabet	1. Readability<br>2. Margin<br>3. Similarity<br>4. Line<br>5. Space<br>6. Size<br>7. Shape<br>8. Slant<br>9. Roundness<br>10. Alignment<br>11. Recognition	Jameel et al., 2017

References:

Amundson, S.J. (1995). *Evaluation Tool of Children's Handwriting.* Homer, AK: OT Kids.

Asselborn, T., Gargot, T., Kidziński, Ł., Johal, W., Cohen, D., Jolly, C., & Dillenbourg, P. (2018). Automated human-level diagnosis of dysgraphia using a consumer tablet. *NPJ digital medicine*, *1*(1), 42. https://doi.org/10.1038/s41746-018-0049-x

Barnett, A. L., Prunty, M., & Rosenblum, S. (2018). Development of the Handwriting Legibility Scale (HLS): A preliminary examination of Reliability and Validity. *Research in Developmental Disabilities, 72*, 240-247.

Barnett, A., Henderson, S., Scheib, B., & Schulz, J. (2007). *Detailed assessment of speed of handwriting. Oxford: Harcourt Assessment.*

Barnett, A., Rosenblum, S., & Prunty, M. (2013). Development of the Handwriting Legibility Scale (HLS): an examination of reliability and validity.*Nara: International Graphonomics Society.*

Bo, J., Colbert, A., Lee, C. M., Schaffert, J., Oswald, K., & Neill, R. (2014). Examining the relationship between motor assessments and handwriting consistency in children with and without probable developmental coordination disorder.*Research in Developmental Disabilities, 35*(9), 2035-2043.

Bumin, G., & Kavak, S. T. (2010). An investigation of the factors affecting handwriting skill in children with hemiplegic cerebral palsy. *Disability and Rehabilitation, 32*(8), 692-703.

Chang, S. H., & Yu, N. Y. (2012). *Chinese Handwriting Evaluation Form: Manual*. Taipei, Taiwan: Psychological Publishing.

Chang, S. H., & Yu, N. Y. (2005). Evaluation and classification of types of Chinese handwriting deficits in elementary schoolchildren. *Perceptual and Motor Skills, 101*(2), 631-647.

Cheng-Lai, A., Li-Tsang, C. W., Chan, A. H., & Lo, A. G. (2013). Writing to dictation and handwriting performance among Chinese children with dyslexia: Relationships with orthographic knowledge and perceptual-motor skills.*Research in Developmental Disabilities, 34*(10), 3372-3383.

Daly, C. J., Kelley, G. T., & Krauss, A. (2003). Relationship between visual-motor integration and handwriting skills of children in kindergarten: A modified replication study. *American Journal of Occupational Therapy, 57*(4), 459-462.

Desai, A. S., & Rege, P. V. (2005). Correlation between developmental test of visual-motor integration [VMI] and handwriting in cerebral palsy children. *The Indian Journal of Occupational Therapy, 37*(2), 27-31.

Duiser, I. H., van der Kamp, J., Ledebt, A., & Savelsbergh, G. J. (2014). Relationship between the quality of children's handwriting and the B eery B uktenica developmental test of visuomotor integration after one year of writing tuition. *Australian Occupational Therapy Journal*, *61*(2), 76-82.

Erez, N., Yochman, A., & Parush, S. (1996). *The Hebrew Handwriting Evaluation.* Jerusalem: The Hebrew University of Jerusalem.

Farhangnia, S., Hassanzadeh, R., & Ghorbani, S. (2020). Handwriting performance of children with attention deficit hyperactivity disorder: The role of visual-motor integration. *International Journal of Pediatrics, 8*(11), 12317-12326.

Gargot, T., Asselborn, T., Pellerin, H., Zammouri, I., M. Anzalone, S., Casteran, L., ... & Jolly, C. (2020). Acquisition of handwriting in children with and without dysgraphia: A computational approach. *PLoS One*, *15*(9), e0237575.

Gilboa, Y., Josman, N., Fattal-Valevski, A., Toledano-Alhadef, H., & Rosenblum, S. (2014). Underlying mechanisms of writing difficulties among children with neurofibromatosis type 1. *Research in Developmental Disabilities, 35*(6), 1310-1316.*

Gilboa, Y., Josman, N., Fattal-Valevski, A., Toledano-Alhadef, H., & Rosenblum, S. (2010). The handwriting performance of children with NF1. *Research in Developmental Disabilities, 31*, 929-935.

Hamstra-Bletz, L., De Bie, J., & Den Brinker, B. P. L. M. (1987). Beknopte beoordelingsmethode voor kinderhandschriften: Experimentele versie [Concise evaluation-scaleforchildren'shandwriting: Experimentalversion]. Lisse: Swets&Zeitlinger.

Havaei, N., Azad, A., ALIZADE, Z. M., & Ebadi, A. (2017). Development and validity of the Persian Handwriting Assessment Tool for primary school-aged children. *Crescent Medical Journal, 19*(3), 1-9.

Hellinckx, T., Roeyers, H., & Van Waelvelde, H. (2013). Predictors of handwriting in children with autism spectrum disorder. *Research in Autism Spectrum Disorders, 7*(1), 176-186.

Jameel, H. T., & Nabeel, T. (2017). Effect of visual motor integration training on legibility of urdu handwriting. *Pakistan Journal of Education, 34*(1).

Jameel, H. T., & Nabeel, T. (2017). Effect of visual motor integration training on legibility of urdu handwriting. *Pakistan Journal of Education, 34*(1).

Kaiser, M. L., Albaret, J. M., & Doudin, P. A. (2009). Relationship between visual-motor integration, eye-hand coordination, and quality of handwriting. *Journal of Occupational Therapy, Schools, & Early Intervention, 2*(2), 87-95.

Klein, S., Guiltner, V., Sollereder, P., & Cui, Y. (2011). Relationships between fine-motor, visual-motor, and visual perception scores and handwriting legibility and speed. *Physical & Occupational Therapy in Pediatrics, 31*(1), 103-114.

Lam, S. S., Au, R. K., Leung, H. W., & Li-Tsang, C. W. (2011). Chinese handwriting performance of primary school children with dyslexia.*Research in developmental disabilities, 32*(5), 1745-1756.

Lee, T. I., Howe, T. H., Chen, H. L., & Wang, T. N. (2016). Predicting handwriting legibility in Taiwanese elementary school children.*American Journal of Occupational Therapy, 70*(6), 1-9.

Linda, F. L., Thanapalan, K. C., & Chan, C. C. (2014). Visual-perceptual-kinesthetic inputs on influencing writing performances in children with handwriting difficulties. *Research in developmental disabilities*, *35*(2), 340-347.

Li-Tsang, C. W., Au, R. K., Chan, M. H., Chan, L. W., Lau, G. M., Lo, T. K., & Leung, H. W. (2011). Handwriting characteristics among secondary students with and without physical disabilities: A study with a computerized tool. *Research in Developmental Disabilities, 32*(1), 207-216.

Li-Tsang, C. W., Li, T. M., Yang, C. N., Leung, H. W., & Zhang, E. W. (2022). Evaluating Chinese handwriting performance of primary school students using the Smart Handwriting Analysis and Recognition Platform (SHARP). *medRxiv.*

Li-Tsang, C. W., Wong, A. S., Leung, H. W., Cheng, J. S., Chiu, B. H., Linda, F. L., & Chung, R. C. (2013). Validation of the Chinese Handwriting Analysis System (CHAS) for primary school students in Hong Kong. *Research in Developmental Disabilities, 34*(9), 2872-2883.

Maeland, A. F. (1992). Handwriting and perceptual-motor skills in clumsy, dysgraphic, and 'normal' children. P*erceptual and Motor Skills, 75*(3_suppl), 1207-1217.

Malloy-Miller, T. (1985). *An Examination of handwriting problem subtypes,* Unpublished Master's Thesis, University of Calgary, Calgary, Alberta.

Mekyska, J., Faundez-Zanuy, M., Mzourek, Z., Galaz, Z., Smekal, Z., & Rosenblum, S. (2016). Identification and rating of developmental dysgraphia by handwriting analysis. *IEEE Transactions on Human-Machine Systems*, *47*(2), 235-248.

Overvelde, A., & Hulstijn, W. (2011). Handwriting development in grade 2 and grade 3 primary school children with normal, at risk, or dysgraphic characteristics. *Research in Developmental Disabilities, 32*(2), 540-548.

Pagliarini, E., Scocchia, L., Vernice, M., Zoppello, M., Balottin, U., Bouamama, S., ... & Stucchi, N. (2017). Children's first handwriting productions show a rhythmic structure. *Scientific reports*, *7*(1), 5516.

Parush, S., Lifshitz, N., Yochman, A., & Weintraub, N. (2010). Relationships between handwriting components and underlying perceptual-motor functions among students during copying and dictation tasks. *OTJR: Occupation, Participation and Health, 30*(1), 39-48.

Preminger, F., Weiss, P. L., & Weintraub, N. (2004). Predicting occupational performance: Handwriting versus keyboarding. *The American Journal of Occupational Therapy, 58*(2), 193-201.

Prunty, M., Barnett, A. L., Wilmut, K., & Plumb, M. (2016). Visual perceptual and handwriting skills in children with Developmental Coordination Disorder.*Human Movement Science, 49*, 54-65.

Reisman, J. (2004). Minnesota Handwriting Assessment. San Antonio, TX: Psychological Corporation Retrieved from http://www.pearsonclinical.com/therapy/products/100000275/minnesota-handwriting-assessment.html.

Rosenblum, S. (2008). Development, reliability, and validity of the Handwriting Proficiency Screening Questionnaire (HPSQ). *The American Journal of Occupational Therapy, 62*(3), 298-307.

Rosenblum, S. (2008). Development, reliability, and validity of the Handwriting Proficiency Screening Questionnaire (HPSQ). *The American Journal of Occupational Therapy, 62*(3), 298-307.

Rosenblum, S., Parush, S., & Weiss, P. L. (2003). Computerized temporal handwriting characteristics of proficient and non-proficient handwriters. *American Journal of Occupational Therapy, 57*, 129-138.

Seyyedrezaei, S. H., Khajeaflaton, S., Ghorbani, S., & Dana, A. (2021). Relative age effects on children's handwriting: Role of visual-motor integration. *International Journal of Pediatrics, 9*(1), 12775-12783.

Shen, I. H., Lee, T. Y., & Chen, C. L. (2012). Handwriting performance and underlying factors in children with attention deficit hyperactivity disorder. *Research in Developmental Disabilities, 33*(4), 1301-1309.

Shih, H. N., Tsai, W. H., Chang, S. H., Lin, C. Y., Hong, R. B., & Hwang, Y. S. (2018). Chinese handwriting performance in preterm children in grade 2. *PloS One, 13*(6), e0199355.

Taverna, L., Tremolada, M., Tosetto, B., Dozza, L., & Renata, Z. S. (2020). Impact of psycho-educational activities on visual-motor integration, fine motor skills and name writing among first graders: a kinematic pilot study. *Children, 7*(4), 27.

Tseng, M. H. (1993). Factorial validity of the Tseng Handwriting Problem Checklist. *Journal of Occupational Therapy Association, 11*, 13-27.

Tseng, M. H., & Murray, E. A. (1994). Differences in perceptual-motor measures in children with good and poor handwriting. *The Occupational Therapy Journal of Research, 14*(1), 19-36.

Van Hartingsveldt, M. J., Cup, E. H., Hendriks, J. C., de Vries, L., de Groot, I. J., & Nijhuis-van der Sanden, M. W. (2015). Predictive validity of kindergarten assessments on handwriting readiness. *Research in Developmental Disabilities, 36*, 114-124.

Van Waelvelde, H., De Mey, B., & Smits-Engelsman, B. C. M. (2009). Systematische Opsporing van Schrijfmotorische stoornissen (SOS).

Van Waelvelde, H., Hellinckx, T., Peersman, W., & Smits-Engelsman, B. C. M. (2012). SOS: A screening instrument to identify children with handwriting impairments. *Physical & Occupational Therapy in Pediatrics, 32*(3), 306–319.

Volman, M. J. M., van Schendel, B. M., & Jongmans, M. J. (2006). Handwriting difficulties in primary school children: A search for underlying mechanisms. *American Journal of Occupational Therapy, 60*(4), 451-460.

Weil, M. J., & Cunningham Amundson, S. J. (1994). Relationship between visuomotor and handwriting skills of children in kindergarten. *The American Journal of Occupational Therapy, 48*(11), 982-988.

Wicki, W., Lichtsteiner, S. H., Geiger, A. S., & Müller, M. (2014). Handwriting fluency in children. *Swiss Journal of Psychology, 73*(2), 87-96.

Yochman, A., & Parush, S. (1998). Differences in Hebrew handwriting skills between Israeli children in second and third grade. *Physical & Occupational Therapy in Pediatrics*, *18*(3-4), 53-65.
