# Supplementary material for: Reliability, validity, and measurement invariance of a Chinese handwriting legibility scale among primary students in central China
Source: Front Psychol. 2023 Jul 28;14:1050894. doi: 10.3389/fpsyg.2023.1050894 (PMC10422027; doi:10.3389/fpsyg.2023.1050894)
Supplement: Supplementary file 2 [file Data_Sheet_2.pdf]

Supplementary material 2:
A - for expert rating of content validity:

Rating form for the Chinese Handwriting Legibility Scale (CHLS)

Name of student:	Class information:
Name of expert:	Profession:

About the background:

The logographic nature and visual-spatial properties of Chinese characters determine the uniqueness and complexity of Chinese handwriting. Combined with the spatial-oriented characteristics of the legibility dimension, the spatial-related nature of Chinese handwriting legibility is further strengthened. In other words, spatial thinking is implicit in legible Chinese character writing, and there is a potential association between Chinese handwriting legibility and students' spatial cognition. However, handwriting evaluation affording an in-depth analysis of spatial aspects of the legibility dimension in the Chinese context is rare.

In this context, with 10 spatial criteria (see details in next page) of handwriting legibility at both the analytic and holistic level, the CHLS aims to provide a detailed analysis of Chinese primary school students' Chinese handwriting legibility performance in central China. A Likert scale ranging from 1 (poor) to 5 (good) is used in the evaluation, with higher scores indicating better legibility. The total score is computed by summing the rating of all criteria.

Chinese primary school students aged 8–12 from the central region, who have undergone several years of Chinese handwriting training and should have developed certain written communication skills, would copy a Chinese template (see first three lines of the Chinese template below) as legibly as possible within 4 mins. The CHLS is used to assess the students' handwriting products.

Please review the CHLS with the rating examples, and record your remarks on the following concerns:

1. The clarity of each criterion:
2. The content breadth:
3. The degree to which you think each criterion contributes to the construct of 'Chinese handwriting legibility':

4. Any additional remarks on the CHLS:

Note:

1. The **analytic-level** evaluation approach focuses on judging or grading the quality of stroke-level (i.e., *within* character) handwriting features according to predetermined standards. By contrast, the **holistic-level** evaluation approach focuses on assessing the character-level (i.e., *between* characters) features of a written passage as compared with a group of pre-graded writing samples.
2. The instructions for scoring h1, h5, h7, h8, h9, and h10 emphasize decision at the analytic level, whereas those for scoring h2, h3, h4, and h6 correspond with the legibility performance at the holistic level.

h1: spacing/spatial relationships between *strokes/radicals* - An overall impression of spacing/spatial relationships between strokes/radicals (i.e., incorrect spacing between strokes/ radicals, incorrect position of components, e.g., overlapping or too far apart, or incorrect junction, collisions, and adhesions).

1 – Poor

5 – Good

1 2 3 4 5

h2: spacing/spatial relationships between *characters* - An overall impression of spacing/spatial relationships between characters (e.g., irregular (too much or no) spaces between characters - characters should be evenly separated).

1 – Poor

5 – Good

1 2 3 4 5

h3: alignment of characters - An overall impression of the alignment of characters (e.g., poor alignment among characters, the row/column lines of writing are slanted from horizontal/vertical orientation).

1 – Poor

5 – Good

1 2 3 4 5

h4: baseline orientation - An overall impression of the baseline orientation (e.g., out of grid/line, overshooting or undershooting the baseline, and inappropriate margins).

1 – Poor

5 – Good

1 2 3 4 5

h5: uniformity of *stroke/radical* size - An overall impression of the uniformity of stroke/radical size (e.g., part of the character is too big or too small; the length of a particular stroke is inappropriate).

1 – Poor

5 – Good

1 2 3 4 5

h6: uniformity of *character* size - An overall impression of the uniformity of character size (e.g., some characters are small, and some characters are big; irregularity of character height).

1 – Poor

5 – Good

1 2 3 4 5

h7: number of strokes - An overall impression of the number of strokes (i.e., superfluous, or missing strokes).

1 – Poor

5 – Good

1 2 3 4 5

h8: closure of stroke/radical - An overall impression of the closure of stroke/radical (i.e., improper closure of stroke/radical parts).

1 – Poor

5 – Good

1 2 3 4 5

h9: line quality - An overall impression of the line quality of strokes (e.g., curves are angular, or straight lines are wavy).

1 – Poor
5 – Good

1	2	3	4	5

h10: direction - An overall impression of the direction of strokes/radicals (e.g., the stroke/radical is not oriented in the correct direction, horizontal/vertical lines are skewed).
1 – Poor
5 – Good

1	2	3	4	5

Sum score: ___________________

B - First three lines of the Chinese template:

工	出	那	到	要	高	球	痛	境
不	奶	作	定	重	院	圈	道	德
文	永	但	些	相	留	集	想	影
